# Self-Medication in Rural Northeastern Romania: Patients’ Attitudes and Habits

**DOI:** 10.3390/ijerph192214949

**Published:** 2022-11-13

**Authors:** Razvan-Nicolae Rusu, Daniela-Carmen Ababei, Walther Bild, Ioana Stoian, Ioana Macadan, Gabriela Dumitrita Stanciu, Andrei Ciobica, Veronica Bild

**Affiliations:** 1 Department of Pharmacodynamics and Clinical Pharmacy, “Grigore T. Popa” University of Medicine and Pharmacy, 16 Universitatii Street, 700115 Iasi, Romania; 2 Advanced Research and Development Center for Experimental Medicine (CEMEX), “Grigore T. Popa” University of Medicine and Pharmacy, 16 Universitatii Street, 700115 Iasi, Romania; 3 Department of Physiology, “Grigore T. Popa” University of Medicine and Pharmacy, 700115 Iasi, Romania; 4 Center of Biomedical Research of the Romanian Academy, 700506 Iasi, Romania

**Keywords:** self-medication, drugs, healthcare, rural, pharmacist, antibiotics, prescription, questionnaire

## Abstract

In recent years, many healthcare systems, along with healthcare professionals, have provided services in a patient-centered manner, in which patients are key actors in the care process. Encouraging self-care creates responsible patients, but it must be practiced responsibly. This study aims to analyze the tendency towards self-medication for patients from a rural area in Northeastern Romania. Data were collected using a questionnaire, which consisted of 25 questions, that has been developed by the research team. Student’s *T* test or one-way ANOVA was used, and the reliability of the questionnaire was calculated using Cronbach’s alpha coefficient. Fifty-eight patients agreed to participate and were interviewed. The results of the study suggest that respondents practice self-medication, which they resort to when their condition cannot be treated with natural remedies or herbs and when it impairs their ability to do their daily activities. Self-medication could be explained by the lack of self-care services as well as the trust patients have in the specific treatment. Patients prefer asking the pharmacist for drugs instead of visiting a physician, which could be due to higher accessibility and time-efficiency, while also being prone to stock up on certain medications due to limited access to healthcare.

## 1. Introduction

In recent years, many healthcare systems, along with healthcare professionals, have focused their attention on providing patient-centered care in a manner in which patients are key actors in the process [1]. The professional’s main task is to ensure that proper health-related recommendations are given to the patient and that medicine is correctly prescribed and administered to and by the patient, while the patient’s role is to practice self-care responsibly, making sure that the required lifestyle changes are met [2].

The concept of self-care is defined by the World Health Organization (W.H.O.) as “the ability of individuals, families, and communities to promote health, prevent disease, maintain health, and to cope with illness and disability with or without the support of a health-care provider” [3].

Encouraging self-care creates empowered and responsible patients who can work together with pharmacists, physicians or other health professionals, thus developing a healthy partnership [4]. For this to be effectively materialized, patients must be aware of their responsibility while also having proper levels of health literacy as well as high trust in their healthcare providers, with trust being considered an indicator of the quality of the healthcare process [5]. An important component of the self-care process is the self-administration of drugs, also known as self-medication, which is considered to be “the taking of drugs, herbs or some remedies on personal initiative, or on the advice of another person, without consulting a physician for the ailment of self-diagnosed physical illness” [6].

Self-medication practices have increased on a global level as a result of economic, cultural and political factors, with reports showing that self-medication prevalence in developing countries ranges from 12.7% to 95% [7]. In regard to the European region, the prevalence of self-medication is estimated at about 68% [8].

The reasons behind the practice of self-medication could be the lack of time to visit a healthcare provider, a reduced number of health institutions in different regions, difficulty in making an appointment to see a specialist or the high cost of consultations [6].

While it can be beneficial when done responsibly, it could also be detrimental to patients’ health due to possible negative consequences [9].

Benefits include reduced visits to the physician, cost savings and time efficiency (e.g., physicians would have more time to devote to more severe cases). It seems that patients consider access to nonprescription medicine to be more convenient than visiting a doctor [10]. Other benefits are higher patient independence, with the final outcome being reduced pressure on medical services [11,12]. Negative aspects include an incorrect diagnosis, delay in seeking appropriate care and an increased risk of drug abuse [12]. Another important problem refers to drug interactions, which have been defined as the modification of the therapeutic effect and/or toxicity of a drug when administered at the same time as either other drugs or food [13]. Patients might not be aware that certain foods/beverages (e.g., grapefruit juice) can alter the pharmacokinetics of drugs, while different drugs taken together can lead to increased toxicity [13,14]. This is especially important in elderly patients, who usually take several drugs due to polypathology and who have certain modified physiological parameters (e.g., reduced body water content, hepatic drug clearance and renal drug elimination) [13].

Patients must possess health literacy skills, which have been defined as “the cognitive and social skills which determine the motivation and ability of individuals to gain access to, understand, and use information in ways which promote and maintain good health” [15]. Low health literacy has been linked to a higher prevalence of long-term health conditions, a reduced ability to interpret labels and health messages, an inability to appropriately administer drugs and higher mortality in older people [16]. Studies show that patients who were incorrectly administering their nonprescription medication stated that they did not know the appropriate duration of treatment, nor the maximum dosage, having not read the label [10].

Ways to improve patients’ health literacy include using simple, non-technical language, reducing written information, and asking them if recommendations have been understood. Patients should also be provided with information regarding risks, benefits and different options for their condition [17].

Patients usually resort to self-medication for situations such as headaches, fever, cough, menstrual pain, eye and skin diseases, sexually transmitted diseases and respiratory tract infections [4]. Some of these conditions can be treated without contacting a physician, but by doing so, a potentially severe underlying pathology can be masked [13].

The issue of self-medication in Romania, especially in the Northeastern region, is important due to the limited number of existing studies [18,19], which mainly focus on the use of NSAIDS/analgesics or antibiotics, in a wide range of patients (urban region/rural region/university students).

The aim of our study was to assess the tendency towards self-medication among people from the rural side of Northeastern Romania, as well as to highlight the possible reasons for this. By doing so, a better understanding of what drives patients towards self-medication could be gained, thus leading to possible improvements in limiting the potential dangers. Observing specific patterns would make room for tailored interventions that could ultimately lead to improving the health status of patients.

## 2. Materials and Methods

The current research is an observational-type study that took place on the rural side of Iași county (Gorban village), Northeastern Romania, which is at the border between two counties, located 54 and 30 km away from 2 main cities and 15 km away from a semi-urban area. It is also in the proximity of the border-crossing point with the Republic of Moldova, and there is only one pharmacy and one primary care physician in the village. There are approximately 2940 residents in the village, most of whom are Romanians, with no particular health conditions that are specific to the area. The main religion is Orthodox-Christian, and the majority of people are retired and have a low-to-middle income. Data were collected using a questionnaire that consisted of 25 questions.

Inclusion criteria: patients who entered the pharmacy in which the study took place and accepted to be a part of this research.

Exclusion criteria: pediatric patients and patients with cognitive impairment.

The questionnaire was developed by a team of researchers comprising pharmacists, a psychologist and a physician, and it was adapted for patients who reside on the rural side of Northeastern Romania. Interviewed patients either attended the local physician’s office, who came more frequently to the pharmacy, or travelers who crossed the border point.

The 25 questions could be grouped into 3 sections. The sections discussed in the current paper refer to basic demographic aspects, as well as factors related to self-medication (e.g., How often do you buy nonprescription drugs?/Do you inform your pharmacist regarding other treatments you are following?). The results of the last section regarding the level of patient trust in the quality of health services are not presented in this work.

The pre-phase of the test started in July 2021 and was conducted on 33 patients selected by random sampling from the target population. After this step, the final version of the questionnaire was obtained and applied between March and June 2022. A final-year pharmacy student was trained to interview the patients and fill out the questionnaire.

The approximate time for answering the questions was 15 min. It was explained to the patients that none of their personal information would be used and that the answers were anonymous. Since participation was voluntary, patients could refuse to respond or end the interview at any moment they wished. This study was approved by the Ethics Committee of “Grigore T. Popa” Iași University of Medicine and Pharmacy (March 2020).

The questions that referred to the frequency of self-medication were assessed using a 4-point Likert scale. In order to obtain a total score for the self-medication scale, we combined the 10 relevant items (items 1, 2, 3, 4, 5, 6, 7, 8, 10, and 13; all 10 items were on a 4-point Likert scale) and assigned a score of 1 to the least-self-medicated answer and a score of 4 to the most relevant one. The resulting sum was a new variable named the total self-medication score. Items that referred to certain drugs that were used by patients (e.g., Did you ever take codeine without consulting a physician or a pharmacist?), as well as those regarding the consumption of tea, coffee, alcohol or tobacco, were yes/no-type questions. Afterwards, we analyzed the results to determine whether the total self-medication score was influenced by any of our independent variables (age groups, biological gender, education, ampicillin use, amoxicillin/clavulanic acid use (which will be referred to as Augmentin), clarithromycin use, sulfamethoxazole/trimethoprim use (which will be referred to as Biseptol), codeine use, metamizole use (which will be referred to as Algocalmin), paracetamol use, cough syrup use, omeprazole use, eye drop use, herpes medication use, use of fever medication for children, use of antibiotics for children, tea consumption, coffee consumption, alcohol consumption and smoking status) using Student’s *T* test in SPSS or the one-way ANOVA if the dependent variable had more than 2 levels. Data were summarized using the mean ($\bar{x}$) and standard deviation (SD). *p*-values less than 0.05 were considered statistically significant. The reliability of the self-medication subscale was calculated using Cronbach’s alpha coefficient.

## 3. Results

The 10-item self-medication subscale has a Cronbach’s alpha coefficient in the acceptable range (0.7–0.8), indicating that this scale is reliable (α = 0.713/Cronbach’s Alpha Based on Standardized Items—α = 0.690).

Our group consisted of 58 patients, 21 males (36.2%) and 37 females (63.8%). In regard to age groups, there were 10 patients (17.2%) between the ages of 18 and 30, 5 patients (8.6%) between 31 and 40, 10 patients (17.2%) between 41 and 50, 12 patients (20.7%) between 51 and 60, 10 patients (17.2%) between 61 and 70, and 11 patients (19%) older than 71 years. Of these, 6 patients (10.3%) went to primary school, 9 patients (15.5%) went to middle school, 4 patients (6.9%) went to trade school, 19 patients (32.8%) went to high school, 9 patients (15.5%) went to post-secondary school, 6 patients (10.3%) had a Bachelor’s degree, and 5 patients (8.6%) had a Master’s degree. The demographic aspects are summarized in Table 1.

In regard to the practice of self-medication, data are presented in Table 2, while aspects regarding the consumption of tea, coffee, alcohol or tobacco can be found in Table 3.

**Figure 1:**
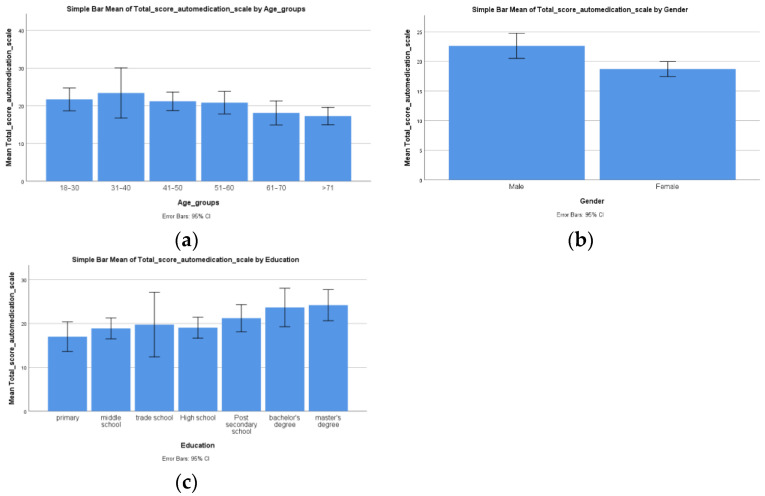
Socio-demographic aspects: Differences regarding the total score of self-medication scale for (**a**) every age group, (**b**) biological gender, and (**c**) every education group.

The one-way ANOVA showed that there was a statistically significant difference (*p* = 0.039) between the age groups of the participants in regard to the total score on the self-medication scale (F (5, 52) = 2.550) (Figure 1a).

Student’s *T* test demonstrated that there was a statistically significant difference (*p* = 0.001) between the female and male patients in regard to the total score on the self-medication scale (F (1, 56) = 12.180), in the sense that males presented a higher total score on the self-medication scale ($\bar{x}$ = 22.62, SD = 4.64) compared to females ($\bar{x}$ = 18.70, SD = 3.78) (Figure 1b).

Our next one-way ANOVA showed that there was a statistically significant difference (*p* = 0.032) between the seven education groups of the patients in regard to the total score on the self-medication scale (F (6, 51) = 2.531) (Figure 1c).

**Figure 2:**
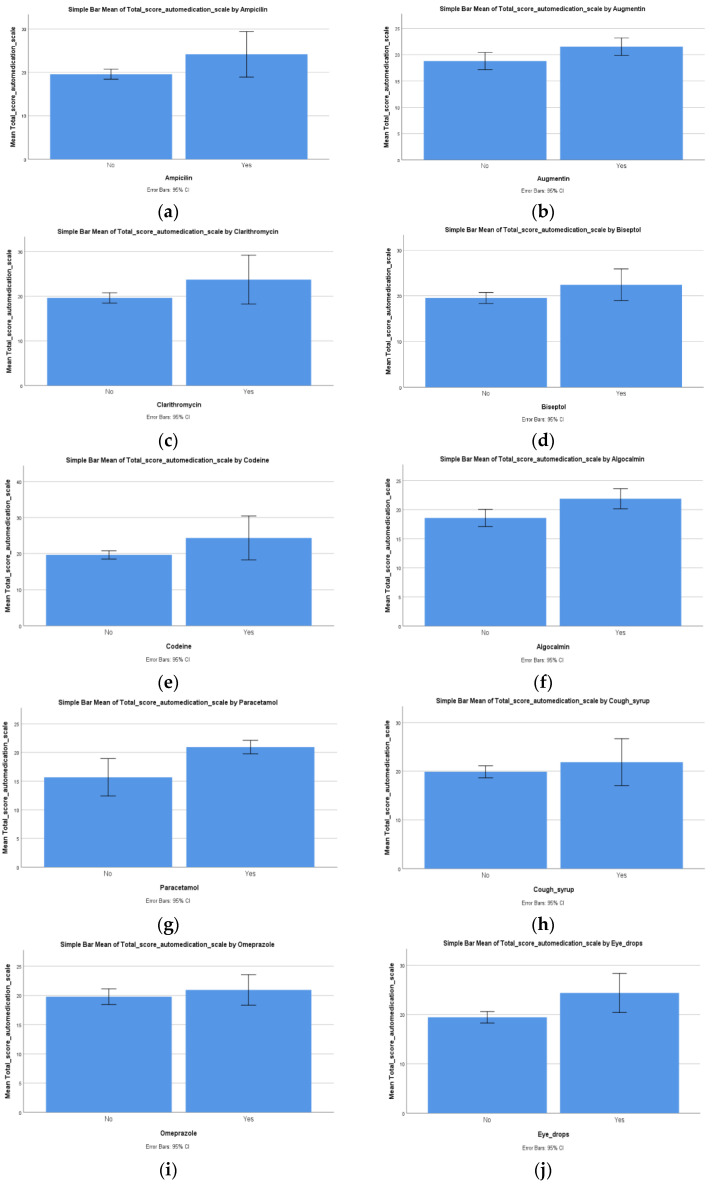

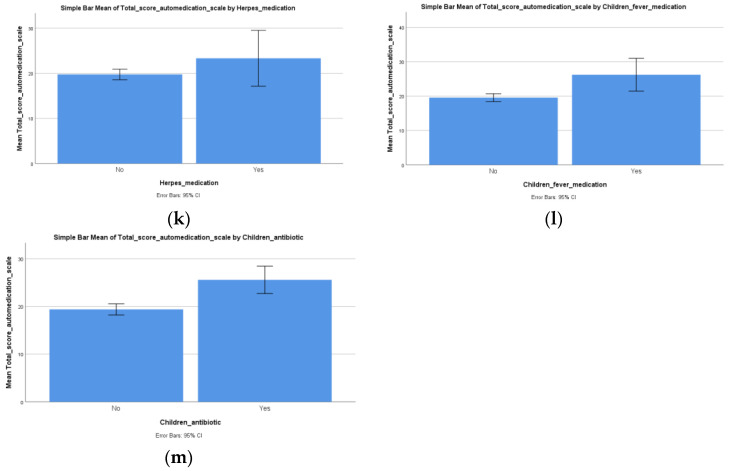
Self-medication aspects: Differences regarding the total score on self-medication scale between the two (**a**) ampicillin-use groups, (**b**) Augmentin-use groups, (**c**) clarithromycin-use groups, (**d**) Biseptol-use groups, (**e**) codeine-use groups, (**f**) Algocalmin-use groups, (**g**) paracetamol-use groups, (**h**) cough-syrup-use groups, (**i**) omeprazole-use groups, (**j**) eye-drop-use groups, (**k**) herpes-medication-use groups, (**l**) fever medication for children use groups, and (**m**) antibiotics for children use groups.

Our next Student’s *T* test demonstrated that there was a statistically significant difference (*p* = 0.010) between patients who used ampicillin and patients who did not use it in regard to the total score on the self-medication scale (F (1, 56) = 7.061), in the sense that patients who used ampicillin had a higher total score on the self-medication scale ($\bar{x}$ = 24.14, SD = 5.66) compared to patients who did not use ampicillin ($\bar{x}$ = 19.57, SD = 4.07) (Figure 2a). This was also demonstrated for Augmentin users and non-users (*p* = 0.019) in regard to the total score on the self-medication scale (F (1, 56) = 5.827). It seems that users had a higher total score on the self-medication scale ($\bar{x}$ = 21.54, SD = 4.27) compared to patients who did not use Augmentin ($\bar{x}$ = 18.80, SD = 4.35) (Figure 2b).

Regarding clarithromycin, our Student’s *T* test demonstrated that there was a statistically significant difference (*p* = 0.023) between users and non-users in regard to the total score on the self-medication scale (F (1, 56) = 5.497). Patients who used clarithromycin had a higher total score on the self-medication scale ($\bar{x}$ = 23.71, SD = 5.90) compared to patients who did not use clarithromycin ($\bar{x}$ = 19.63, SD = 4.09) (Figure 2c).

Our next Student’s *T* test demonstrated that there was a statistically significant difference (*p* = 0.046) between patients who used Biseptol and patients who did not use it in regard to the total score on the self-medication scale (F (1, 56) = 4.173). The reported significant differences between the two samples of patients showed that the patients who used Biseptol had a higher total score on the self-medication scale ($\bar{x}$ = 22.42, SD = 5.45) compared to non-users ($\bar{x}$ = 19.52, SD = 4.06) (Figure 2d).

Regarding codeine use, our next Student’s *T* test demonstrated that there was a statistically significant difference (*p* = 0.014) between users and non-users in regard to the total score on the self-medication scale (F (1, 56) = 6.448). The reported significant differences showed that patients who used codeine had a higher total score on the self-medication scale ($\bar{x}$ = 24.33, SD = 5.82) compared to patients who did not use codeine ($\bar{x}$ = 19.63, SD = 4.11) (Figure 2e).

Our next Student’s *T* test demonstrated that there was a statistically significant difference (*p* = 0.004) between patients who used Algocalmin and patients who did not use it in regard to the total score on the self-medication scale (F (1, 56) = 8.194). The differences showed that patients who used Algocalmin had a higher total score on the self-medication scale ($\bar{x}$ = 21.89, SD = 4.38) compared to patients who did not use Algocalmin ($\bar{x}$ = 18.58, SD = 4.04) (Figure 2f).

In regard to paracetamol, our next Student’s *T* test demonstrated that there was a statistically significant difference (*p* = 0.001) between patients who used paracetamol and patients who did not use it in regard to the total score on the self-medication scale (F (1, 56) = 12.606). The reported significant differences between the two samples of patients showed that patients who used paracetamol had a higher total score on the self-medication scale ($\bar{x}$ = 20.94, SD = 4.06) compared to patients who did not use paracetamol ($\bar{x}$ = 15.67, SD = 4.24) (Figure 2g).

Our next Student’s *T* test demonstrated that there was a non-statistically significant difference (*p* = 0.279) between cough syrup users and non-users in regard to the total score on the self-medication scale (F (1, 56) = 1.194). The reported differences between the two samples of patients showed that patients who used cough syrup had a higher total score on the self-medication scale ($\bar{x}$ = 21.86, SD = 5.21) compared to patients who did not use cough syrup ($\bar{x}$ = 19.88, SD = 4.38), but the measured differences did not reach statistical significance (Figure 2h).

This was also observed for the use of omeprazole, for which our Student’s *T* test revealed a non-significant difference (*p* = 0.375) between patients who used the drug and non-users in regard to the total score on the self-medication scale (F (5, 52) = 0.800). The reported differences showed that patients who used omeprazole had a higher total score on the self-medication scale ($\bar{x}$ = 20.94, SD = 5.08) compared to patients who did not use omeprazole ($\bar{x}$ = 19.78, SD = 4.24), but these observed differences were not statistically significant (Figure 2i).

Our next Student’s *T* test demonstrated that there was a statistically significant difference (*p* = 0.003) between patients who used eye drops and patients who did not use them in regard to the total score on the self-medication scale (F (1, 56) = 9.576); the differences showed that users had a higher total score on the self-medication scale ($\bar{x}$ = 24.38, SD = 4.74) compared to non-users ($\bar{x}$ = 19.44, SD = 4.10) (Figure 2j).

The following Student’s *T* test showed that there was a statistically non-significant difference (*p* = 0.064) between patients who used herpes medication and patients who did not use it in regard to the total score on the self-medication scale (F (1, 56) = 3.578). The reported differences between the two samples of patients showed that patients who used herpes medication had a higher total score on the self-medication scale ($\bar{x}$ = 23.33, SD = 5.88) compared to patients who did not use herpes medication ($\bar{x}$ = 19.75, SD = 4.21). However, these differences were not statistically significant (Figure 2k).

The next Student’s *T* test demonstrated that there was a statistically significant difference (*p* = 0.001) between patients who used fever medication for children and patients who did not use it in regard to the total score on the self-medication scale (F (1, 56) = 11.947). The reported significant differences showed that patients who used fever medication for children had a higher total score on the self-medication scale ($\bar{x}$ = 26.20, SD = 3.83) compared to patients who did not use fever medication for children ($\bar{x}$ = 19.55, SD = 4.13) (Figure 2l).

The following Student’s *T* test demonstrated that there was a statistically significant difference (*p* < 0.0001) between patients who used antibiotics for children and patients who did not use them in regard to the total score on the self-medication scale (F (1, 56) = 14.497). These differences showed that the patients who used antibiotics for children had a higher total score on the self-medication scale ($\bar{x}$ = 25.57, SD = 3.10) compared to patients who did not use antibiotics for children ($\bar{x}$ = 19.37, SD = 4.13) (Figure 2m).

**Figure 3:**
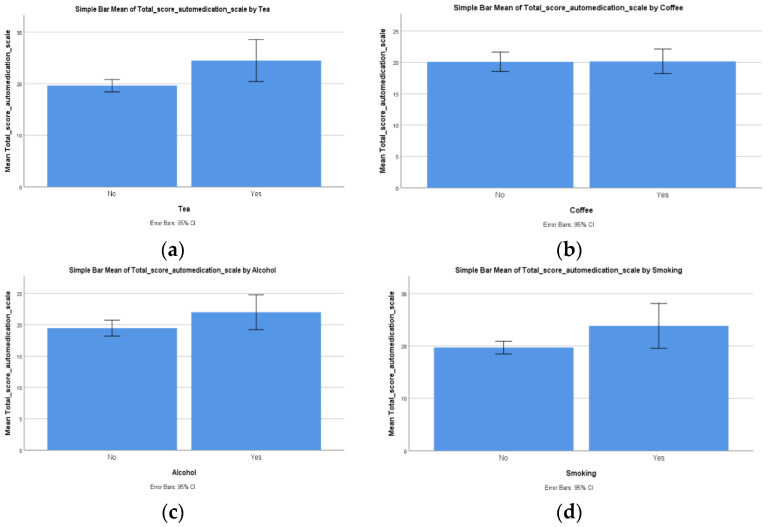
Differences regarding the total score of self-medication scale between the two (**a**) tea consumption groups, (**b**) coffee consumption groups, (**c**) alcohol consumption groups and (**d**) smoking habit groups.

Our next Student’s *T* test demonstrated that there was a statistically significant difference (*p* = 0.010) between patients who consumed tea and patients who did not in regard to the total score on the self-medication scale (F (1, 56) = 7.034), and it seems that consumers had a higher total score on this scale ($\bar{x}$ = 24.50, SD = 3.88) compared to non-consumers ($\bar{x}$ = 19.62, SD = 4.30) (Figure 3a).

Our next Student’s *T* test demonstrated that there was a statistically non-significant difference (*p* = 0.954) between patients who consumed coffee and patients who did not consume it in regard to the total score on the self-medication scale (F (1, 56) = 0.003). The reported differences showed that patients who consumed coffee had a higher total score on the self-medication scale ($\bar{x}$ = 20.16, SD = 4.74) compared to patients who did not consume coffee ($\bar{x}$ = 20.09, SD = 4.36), but these differences were not significant from a statistical point of view (Figure 3b).

The following Student’s *T* test demonstrated that there was a non-significant difference (*p* = 0.059) between patients who consumed alcohol and patients who did not consume it in regard to the total score on the self-medication scale (F (1, 56) = 3.710). The reported differences between the two samples of patients showed that patients who consumed alcohol had a higher total score on the self-medication scale ($\bar{x}$ = 22, SD = 5) compared to patients who did not consume alcohol ($\bar{x}$ = 19.47, SD = 4.16); however, these differences were not significant (*p* > 0.05) (Figure 3c).

Our last Student’s *T* test demonstrated that there was a statistically significant difference (*p* = 0.031) between patients who smoked and patients who did not smoke in regard to the total score on the self-medication scale (F (1, 56) = 4.883). These significant differences showed that patients who smoked had a higher total score on the self-medication scale ($\bar{x}$ = 23.83, SD = 4.07) compared to patients who did not smoke ($\bar{x}$ = 19.69, SD = 4.37) (Figure 3d).

## 4. Discussion

This study aimed to evaluate the practice of self-medication for conditions for which patients often request nonprescription or prescription drugs. As mentioned above, there was only one medical office in the locality with a single physician to whom all people were referred, and this lack of health services has been considered one of the factors that contribute to self-medication practice [20].

It seems that, most often, patients requested medication for the common cold or symptoms that resemble it, for digestive conditions with acute manifestations, for dermatological and ocular problems, for musculo-skeletal conditions and for different infections. For pediatric patients, carers usually asked for fever-lowering medication, cough preparations and antibiotics. Considering the above-mentioned aspects, the research team concluded that drugs that should be monitored in regard to self-medication practices due to their frequent use are ampicillin, amoxicillin/clavulanic acid, clarithromycin, sulfamethoxazole/trimethoprim, acyclovir, ocular preparations with antibiotics, analgesics, antipyretics, metamizole, paracetamol, omeprazole, and central- and peripheral-acting antitussive agents for both children and adults.

It was shown that, during this study, patients often requested ampicillin for acute upper respiratory tract infections (e.g., amigdalitis and tracheitis), otitis, Staphylococcus-associated skin infections (for infected wounds or stings), nail infections and dental infections. Ampicillin is a well-known drug and has been used for a long time in Romania, and most patients are familiar with it. Its indications include infections of the respiratory tract, bacterial meningitis, endocarditis, septicemia and gastrointestinal and genitourinary infections [21]. It is commonly used in Romania, with many patients using it at least once. Advice from family members or friends, as well as the positive effects observed by patients after using it, led to high levels of trust regarding its use. Therefore, misuse is not uncommon. One of the main problems that could derive from this is resistance, a problem that was highlighted in the early days of penicillin use, as stated by Alexander Fleming himself: “It is not difficult to make microbes resistant to penicillin in the laboratory by exposing them to concentrations not sufficient to kill them, and the same thing has occasionally happened in the body” [22].

Easy access to as well as high trust in antibiotics such as ampicillin could lead to misuse and increased resistance, and difficulties especially arise in the case of patients who do not have a prescription and solicit an antibiotic for use until visiting a physician. Considering that the village in which the study took place has only one primary care physician without a working schedule on weekend days, the pharmacist should dispense antibiotics to patients until they can visit the physician. This could mean a 3-day treatment, or even more. According to the deontological code of the pharmacist, prescription-only drugs can be dispensed to patients without a prescription for a treatment of 72 h if they can prove they are under treatment and need to administer that drug, but not for more than 3 days [23].

Another drug that was often requested was the combination of amoxicillin and clavulanic acid (a beta-lactamase inhibitor) (Augmentin), which is one of the most used antimicrobials worldwide. The combination is used for the treatment of aspiration pneumonia, community-acquired pneumonia, urinary tract infections, skin infections, acute otitis media and acute bacterial rhinosinusitis [24]. Patients from the study region often requested Augmentin for dental conditions and different types of infections, such as upper respiratory tract infections and *Staphylococcus* infections.

Another popular antibiotic was clarithromycin, which belongs to the family of macrolide antibiotics and is used in infections of the upper and lower respiratory tracts, skin infections and *Helicobacter pylori* infections [25,26]. It is preferred by patients who are allergic to ampicillin, as well as those who have had good results after administering erythromycin, which is no longer available. A frequently encountered situation by pharmacists refers to patients asking for clarithromycin in the case of a relapse of *Helicobacter pylori* infection. Patients usually reinitiated therapy without consulting a specialist, even though their initial treatment comprised several drugs (e.g., amoxicillin). This might be explained by the fact that patients are convinced that the treatment is going to help them based on previous experiences.

The combination of sulfamethoxazole and trimethoprim (Biseptol) is a well-known and approved treatment for urinary tract infections, acute infective exacerbation of chronic bronchitis and otitis media in pediatrics [27]. Although it was not often prescribed in the early 2000s compared to other drugs that were considered “new” at the time (e.g., quinolones and macrolides), in recent years, the combination has made a comeback, especially for the treatment of respiratory and urinary tract infections. This absence of prescribing led to a decrease in the levels of bacterial resistance.

Considering that most respondents were patients over the age of 50, many knew of the combination, had taken it at least once and trusted it and were prone to using it again. What is important to note is the possibility of drug-induced immune thrombocytopenia, which is rare but possibly life-threatening [28].

Another aspect that was worth noting relates to their anti-folate effect. Petersen and his colleagues found that moderate-dose trimethoprim exposure for 7 days led to a significant decrease in serum folate levels in healthy patients, which could indicate the first stage of a negative folate balance preceding the depletion of folate from tissues. It is necessary to assess patients’ folate status before treatment with trimethoprim, especially for those at risk of deficiency [29], and if this is the case, supplementation with folic acid could be of use [30].

Acyclovir is used to treat infections with herpes simplex virus. Although it is a commonly used drug, its use did not show statistical significance.

For coughing associated with the common cold or different respiratory tract infections, patients frequently asked the pharmacist for codeine, a central opioid antitussive that acts by binding to mu receptors [31]. Patients resorted to pharmacological treatment only after all natural remedies were exhausted. In the study area, antitussives were more requested compared to expectorants. This can be correlated with patients’ health literacy levels: patients preferred a drug that stops the cough, despite the presence of secretion, thus leading to an accumulation that can favor infections. Patients do not usually take higher doses than recommended, and their trust in this drug is high. Although they might already have a prescription that contains codeine, many are not aware and could ask the pharmacist for other products that also contain codeine. On the rural side, the number of healthcare specialists and institutions is reduced, which could explain the tendency to acquire more than needed. The opioid-like side effects of codeine use are euphoria, constipation and sedation. Another important aspect is related to the abuse potential of the drug, which has been seen in different studies. By repeatedly administering codeine, tolerance can develop, leading to increasing doses and a risk of dependence [32].

Metamizole is one of the strongest non-opioid analgesic drugs, which acts by inhibiting COX-3 and by influencing the endogenous opioidergic system [33]. In Romania, there are no official data and no reported cases of metamizole-induced agranulocytosis [34], a potentially fatal adverse reaction that was discovered in the early 1930s. Studies have shown that the risk of this adverse reaction varies widely across countries, some considering that the incidence is about one case per million inhabitants per year. Thus, the fact that metamizole is a prescription drug is justified, limiting the possibility of agranulocytosis [35]. In Romania, patients use oral or parenteral metamizole for musculoskeletal conditions, biliary or renal colic, dental pain, menstrual pain, dental extractions and surgical services. Despite the fact that the pharmaceutical market has numerous OTC-type analgesic drugs, patients prefer metamizole, and its change from the OTC category to prescription-only was hard to accept by many. The study population used metamizole for pain conditions associated with different types of trauma, rheumatic diseases, dental pain and menstrual pain.

Paracetamol is one of the most famous analgesics and antipyretics, which dominates the OTC analgesic drug market due to its lack of gastrointestinal adverse effects (unlike NSAIDS). It is used for the treatment of acute conditions such as headaches, menstrual and dental pain and different types of musculoskeletal pain [36]. It can be found in numerous pharmaceutical products for the treatment of the common cold, either on its own or combined with other substances for its analgesic/antipyretic action, and although it is an easy-to-acquire OTC drug, abuse can lead to hepatic and renal damage. From this standpoint, the pharmacist is limited to telling the patient the risks of abuse, without being able to refuse its dispense or at least monitor its consumption. A possible explanation for the high levels of paracetamol dispensing could be related to the COVID-19 pandemic, which led to a tendency of patients to stock up on the drug. Based on the World Health Organization pain ladder, paracetamol was considered the first-line antipyretic and analgesic for COVID-19 patients, with WHO recommending it instead of ibuprofen if specific symptoms of COVID-19 were present. These recommendations might be due to the potential protective effect against infection with SARS-CoV-2 that paracetamol is considered to possess [37].

The use of antitussive syrups in children was not statistically significant, and neither was omeprazole, which could probably be explained by the fact that patients are more familiar with ranitidine, a competitive inhibitor of H2 histamine receptors [38]. Since its withdrawal from the pharmaceutical market, patients have focused on other drugs. This differs from city pharmacies, in which patients request omeprazole, even if it is a prescription drug. The data obtained were on patient benefits and did not indicate a tendency towards self-medication. It is indicated in conditions such as peptic ulcer disease, duodenal ulcer disease, *H. pylori* infection and gastroesophageal reflux disease, but its unsupervised use could lead to adverse reactions, especially with long-term use. It is recommended that minimum doses for short periods of time be prescribed [39]. Patients from our study requested omeprazole mostly for gastroduodenal ulcers, as well as for gastroesophageal reflux.

Ocular allergies refer to different conditions associated with conjunctivitis, discharge and itching [40]. Many patients develop such types of conditions, especially in spring and autumn, since the region in which the study took place is on the rural side, and the main occupation of patients is farming, as well as different activities around the household. Thus, patients are exposed to allergic factors.

The use of antifever drugs for pediatric patients was statistically significant. Many of these products are over-the-counter-type drugs, with pharmacists having limited control over what the parent decides to do or how many drugs the parent intends to give to the child. The overuse of such drugs can be a consequence of an overprotective attitude of the parents, while another problem is related to different drug forms that contain the same active substance (e.g., paracetamol), of which parents might not be aware. This, in turn, can lead to overuse and adverse effects. It seems that combinations of acetaminophen and ibuprofen are more effective in reducing fevers compared to monotherapy. While these drugs are considered relatively safe when they are used properly, errors in dosage or overuse are often present, with the most important adverse reactions reported being hepatic injury (for paracetamol) and acute kidney injury and gastrointestinal reactions (for ibuprofen) [41].

Antibiotic use in children without proper counseling was also statistically significant. Quite often, parents put pressure on healthcare providers to prescribe antibiotics, considering this to be the best solution, which can be linked to their health literacy levels as well as their trust in healthcare providers. Even if professionals advise against using antibiotics, parents might feel ignored or scared for their children’s safety, thus trying to convince the physician to prescribe such products. Several studies have reported that perceived pressure from parents is the main reason for antibiotic prescribing in pediatric populations. Clinicians avoid prescribing such drugs when they do not feel pressure from parents, being concerned about the problems of antibiotic resistance and over-prescription [42]. Antibiotic overuse is associated with a series of negative consequences, such as inflammatory bowel diseases, reduced diversification of the microbiota and an increased risk for atopic diseases [43].

On the rural side, patients often use natural remedies for common conditions. Of these, it is important to note different types of tea (e.g., *chamomille*, linden, green tea, *chelidonium* tea, St. John’s Wort and peppermint). Although patients consider them to be safe, some of those listed above may influence the pharmacokinetics and pharmacodynamics of drugs. For example, *Chelidonium majus* tea is used for dyspepsia, biliary disorders and irritable bowel syndrome due to its spasmolytic and choleretic properties. However, its active compounds can influence the activity of cytochrome P450 (CYP) enzymes: sanguinarine inhibits CYP1A1, CYP1A2, CYP3A1, CYP3A4, CYP2C8, CYP2C9, CYP2D1 and CP2E1, which can lead to negative consequences when administered concomitantly with drugs metabolized by the fractions mentioned by increasing the plasma concentration of the drug [44]. Another example is St. John’s Wort tea, which can induce the metabolism of associated drugs, especially those that are a substrate for CYP3A4 [45], as well as peppermint tea, which is frequently consumed in Romania and which has the potential to inhibit drug metabolism [46].

*Matricaria chamomilla* tea is also frequently consumed, and it is important to note that the major components of its essential oils are inhibitors of CYP1A2 and CYP3A4 [47], a fact that might not be known by patients.

For our study population, it seems that patients who consumed more tea were also self-medicating to a greater extent. It is important to note that, generally, tea is considered safe and is one of the first options patients resort to when feeling unwell. Thus, patients use tea while under treatment with different drugs, making them susceptible to interactions.

Another important aspect is that the area in which the study took place is a wine-growing area in which people frequently consume alcohol (especially wine or distilled alcoholic beverages). It is known that alcohol influences the bioavailability of drugs, depending on how it is consumed; acute exposure causes the inhibition of drug metabolism, while chronic consumption leads to the induction of the enzymes responsible for metabolization [48]. People from the study area usually make their own wine in the traditional way, without using preservatives or other substances. An important fact is that, as a general rule, they know that they must not consume alcohol if they are using drugs, especially in the case of antibiotics.

While coffee can interact with drugs through both pharmacokinetic and pharmacodynamic mechanisms, pharmacokinetic ones are responsible for a larger number of such interactions. Coffee influences the absorption process through complex formation as well as gastrointestinal pH changes (e.g., caffeine accelerates the absorption of levodopa). It is also known to induce CYP1A2 enzymatic activity and therefore to influence the metabolism of certain associated drugs [49,50].

Last but not least, tobacco consumption can also lead to a series of interactions due to its capacity to alter the pharmacokinetics of certain drugs. It is known that cigarette smoke can induce CYP isoforms such as CYP1A1, CYP1A2 and possibly CYP2E1. Pharmacodynamic interactions are also possible, with nicotine and its stimulant actions being the incriminating factor. People are usually not aware of the potential of cigarette smoke to cause drug interactions, often considering it less harmful from this standpoint than coffee and alcohol [51].

In our study, tobacco users practiced self-medication to a greater extent. It is important to note that people are not usually aware of the negative consequences tobacco use can have on drug treatments and metabolism.

We observed that while patients were aware that coffee and especially alcohol are not to be taken with drugs since they could pose a danger to their health, the fact that tea could also influence drug metabolism was not as known.

Even though it was not measured in our study and reflects an aspect that is subject to change, by answering the questions, it seemed that the patients who were interviewed did not have a tendency to overuse drugs. In regard to infectious diseases or viral conditions, patients usually try to heal themselves with natural or herbal remedies. If this does not work and their health condition deteriorates, making them unable to perform their daily activities, they usually ask the pharmacist for an antibiotic, since this is more convenient than making an appointment with the primary care physician.

Many patients, especially those over the age of 50, usually request antibiotics for at least 3 days. They are aware that antibiotic treatment must last for a couple of days, which could be favorable in regard to general resistance. However, if their condition does not improve, they usually refer to a physician before continuing the administration.

A problem for the pharmacist is in regard to dispensing prescription drugs to patients who do not have a prescription. The deontological code of the pharmacist notes that there are situations in which this can be done. It is considered that, exceptionally, the pharmacist can dispense such drugs without a prescription for a maximum treatment duration of 72 h while also registering the patient’s identification data. This can be done if the patient is under proven treatment and needs to administer the drug, if the patient needs to initiate or maintain a treatment (which has been confirmed by the physician) or if the patient is in need of drugs and/or first aid [23]. Thus, although specific drugs are not mentioned, one could conclude that pharmacists can decide to dispense any drugs considered necessary for the patient for a maximum of 3 days, based on their knowledge and expertise. If the pharmacist decides not to dispense, the patient might remain without treatment, since there is only one pharmacy and one primary care physician, without night shifts or a weekend work schedule. This limited access to healthcare services could put pressure on the pharmacist to dispense certain drugs.

All of the above-mentioned aspects highlight different reasons for which patients resort to self-medication, even though some of the drugs are prescription-only. By understanding what motivates this act, interventions can be made in order to limit potential dangers that can arise. Two main issues are related to self-medication in this context: the lack of healthcare services and levels of health literacy, which are both contributing factors. Before implementing programs, it is helpful to take note of similar actions in different contexts and by other states. For example, reforms in the health sectors in 2005 in Iran aimed to increase the rural population’s access to secondary healthcare services while also expanding health insurance coverage. As part of those reforms, approximately 6000 physicians and 4000 midwives entered the private healthcare network. The consequence was a large increase in hospitalization rates, from 38 to 67 (per 1000 inhabitants per year), an increase that is likely due to improved access to health services, considering the needs of the previously underserved population [52]. Another positive example could refer to the accreditation process of healthcare centers. Accreditation is considered to be a procedure by which healthcare centers are assessed based on a set of predetermined standards established by an independent professional accrediting body [53]. In Romania, pharmacies are accredited by county commissions, and this process consists of several steps. In regard to human resources, a series of aspects are mentioned, of which the 40 h of professional education (known as continuing pharmaceutical education) is of importance for our topic, since this is a way through which pharmacists are engaged in the process of learning and perfecting their medical act [54]. Especially in small communities, such as the one in which our study took place, patients might have better results with their treatment as well as improved health literacy if appropriate interventions are made. A central role could be played by the pharmacist in this context, since patients would rather visit the pharmacy before going to the doctor for medical advice. This is important because tailored interventions are needed, with a good understanding of specific local aspects, limitations and constraints, which are best known by professionals who serve the specific population in the region. Such interventions need the involvement of both patients and health professionals in order to be effective, and this participation must be stimulated through proper incentivized means. Some authors suggest that important incentive means are derived either from the enabling environment or from policies, institutions and regulations [55].

In Romania, reforms were initiated with the ultimate goal of improving the healthcare process. Of these, we mention the “Health for Prosperity” National Health Strategy, 2014–2020, which provided a framework for ensuring access to health services and health system sustainability. While most of the reforms from this program were oriented towards ensuring health system sustainability and predictability, fewer were directed at ensuring equitable access to health services and improving the health of the population. Thus, specific interventions tailored to the characteristics of patients (which can differ between regions) made by professionals who know the problems their population is facing are much needed [56].

### Limitations

The current study is part of a doctoral research project, and it was conducted during the COVID-19 pandemic, which limited patients’ availability for interviews. This explains the gap between the approval received from the Ethics Committee and the two phases of the test per se, as well as the number of participants. Another important aspect is that, although the study is useful in creating a portrait of patients from the specified region and their self-medication and self-care attitudes, which can lead to tailored interventions for improving this process, questioning patients from different regions might lead to different results, which could suggest that its extrapolation potential is limited.

## 5. Conclusions

The purpose of this study was to highlight self-medication and the context in which it takes place while also linking it to aspects such as age, education, gender, the consumption of coffee, alcohol, tea or tobacco and frequently used drugs in self-medication practice. Even though this study is a pretest phase for the instrument that was used, the questionnaire proved to be a reliable resource that can monitor the tendency of the target population to self-medicate and offer information in this direction.

Although our respondents practice self-medication, it seems that they only resort to it if their condition cannot be treated with natural remedies or herbs. While they are aware that alcohol and coffee should not be consumed when under treatment with drugs, the impact of tea and tobacco is not as known. Patients usually visit the pharmacy and ask for drugs when their condition affects their daily activities. They would rather ask the pharmacist for drugs instead of visiting a physician, and this could be due to higher accessibility and time convenience. At the same time, due to limited healthcare services in the study area, patients are prone to stock up on different drugs.

In Romania, especially in small rural communities that might be underserved and in which healthcare providers have gotten to know their patients really well, interventions for increasing the levels of health literacy as well as access to healthcare services are needed. While increasing access to services might be more difficult to implement, steps towards higher levels of health literacy could be made by professionals from the specific area. They are the ones who know their patients, their challenges and their difficulties and could efficiently suggest and even implement interventions with the ultimate goal of improving health. For this, a tailored approach is needed, since “one-size-fits-all”-type solutions might not lead to the expected results.

## Figures and Tables

**Table 1:** Mean scores and standard deviation for the self-medication scale for different demographic groups.

Variable	Mean (x)	Standard Deviation	*p*-Values
Sex			
Males (36.2%)	22.62	4.64	0.001
Females (63.8%)	18.70	3.78	
Age			
18–30 (17.2%)	21.7	4.218	0.039
31–40 (8.6%)	23.40	5.367	
41–50 (17.2%)	21.20	3.425	
51–60 (20.7%)	20.83	4.707	
61–70 (17.2%)	18.10	4.458	
>71 (19%)	17.27	3.467	
Education			
Primary (10.3%)	17.00	3.225	0.032
Middle school (15.5%)	18.89	3.100	
Trade school (6.9%)	19.75	4.646	
Highschool (32.8%)	19.05	4.961	
Post-secondary school (15.5%)	21.22	4.024	
Bachelor’s (10.3%)	23.67	4.179	
Master’s (8.6%)	24.20	2.864	

**Table 2:** Self-medication data.

Variable	Mean (x)	Standard Deviation	*p*-Values
Self-medication drugs			
Ampicillin			
Users (12.1%)	24.14	5.66	0.01
Non-users (87.9%)	19.57	4.07	
Amoxicillin/Clavulanic acid			
Users (48.3%)	21.54	4.27	0.019
Non-users (58.7%)	18.80	4.35	
Clarithromycin			
Users (12.1%)	23.71	5.90	0.023
Non-users (87.9%)	19.63	4.09	
Sulfamethoxazole/Trimethoprim			
Users (20.7%)	22.42	5.45	0.046
Non-users (79.3%)	19.52	4.06	
Codeine			
Users (10.3%)	24.33	5.82	0.014
Non-users (89.7%)	19.63	4.11	
Metamizole			
Users (46.6%)	21.89	4.38	0.004
Non-users (53.4%)	18.58	4.04	
Paracetamol			
Users (84.5%)	20.94	4.06	0.001
Non-users (15.5%)	15.67	4.24	
Cough syrup			
Users (12.1%)	21.86	5.21	0.279
Non-users (87.9%)	19.88	4.38	
Omeprazole			
Users (29.3%)	20.94	5.08	0.375
Non-users (70.7%)	19.78	4.24	
Eye drops			
Users (13.8%)	24.38	4.74	0.003
Non-users (86.2%)	19.44	4.10	
Herpes medication			
Users (10.3%)	23.33	5.88	0.064
Non-users (89.7%)	19.75	4.21	
Fever medication for children			
Users (8.6%)	26.20	3.83	0.001
Non-users (91.4%)	19.55	4.13	
Antibiotics for children			
Users (12.1%)	25.57	3.10	<0.0001
Non-users (87.9%)	19.37	4.13	

**Table 3:** Tea, coffee, alcohol or tobacco consumption.

Variable	Mean (x)	Standard Deviation	*p*-Values
Tea			
Consumers (10.3%)	24.50	3.88	0.010
Non-consumers (89.7%)	19.62	4.30	
Coffee			
Consumers (56.9%)	20.16	4.74	0.954
Non-consumers (43.1%)	20.9	4.36	
Alcohol			
Consumers (25.9%)	22	5	0.059
Non-consumers (74.1%)	19.47	4.16	
Tobacco			
Consumers (10.3%)	23.84	4.07	0.031
Non-consumers (89.7%)	19.69	4.37	

## Data Availability

Not applicable.
